# Red, Green, and Blue Photoluminescence of Ba_2_SiO_4_:M (M = Eu^3+^, Eu^2+^, Sr^2+^) Nanophosphors

**DOI:** 10.3390/ma6083079

**Published:** 2013-07-24

**Authors:** Huayna Cerqueira Streit, Jennifer Kramer, Markus Suta, Claudia Wickleder

**Affiliations:** Inorganic Chemistry, Department of Science and Technology, University of Siegen, Siegen 57068, Germany; E-Mails: cerqueira.streit@chemie.uni-siegen.de (H.C.S.); kramer@chemie.uni-siegen.de (J.K.); suta@chemie.uni-siegen.de (M.S.)

**Keywords:** barium orthosilicate, divalent europium, nanoparticles, luminescence

## Abstract

Divalent europium doped barium orthosilicate is a very important phosphor for the production of light emitting diodes (LEDs), generally associated to the green emission color of micron-sized crystals synthesized by means of solid-state reactions. This work presents the combustion synthesis as an energy and time-saving preparation method for very small nano-sized Ba_2_SiO_4_ particles, flexibly doped to acquire different emission energies. The size of the resulting spherical nanoparticles (NPs) of the green emitting Ba_2_SiO_4_:Eu^2+^ was estimated to about 35 nm applying the Scherrer equation and further characterized with aid of atomic force microscopy (AFM) as well as scanning electron microscopy (SEM). This phosphor is able to build homogeneous luminescent suspensions and was successfully down-sized without changing the optical properties in comparison to the bulk phosphors. Besides the X-ray diffraction (XRD) analysis and the different types of microscopy, the samples were characterized by luminescence spectroscopy. Undoped Ba_2_SiO_4_ NPs are not luminescent, but show characteristic red emission of the ^5^D_0_ → ^7^F*_J_* (*J* = 0–4) electronic transitions when doped with Eu^3+^ ions. Moreover, these orthosilicate nanoparticles generate blue light at low temperatures due to impurity-trapped excitons, introduced by the partial substitution of the Ba^2+^ with Sr^2+^ ions in the Ba_2_SiO_4_ lattice causing a substantial distortion. A model for the temperature behavior of the defect luminescence as well as for their nature is provided, based on temperature-dependent luminescence spectra and lifetime measurements.

## 1. Introduction

Alkaline earth silicate phosphors activated with Eu^2+^ ions have been extensively studied over the past 40 years due to their excellent thermal stability, water resistance, non-sensitivity to air and extraordinary optical properties. For instance, investigations of the optical properties of Eu^2+^-activated Ba_2_SiO_4_ have been reported by several authors, which highlighted its applications in solid-state lighting, especially for the production of Hg-discharge lamps and LEDs [1,2,3,4,5]. However, the phosphors reported within these works consist of micrometer-sized crystals. These so-called bulk phosphors are usually prepared by means of solid state reactions, requiring high temperatures and long reaction time. The resulting enlarged crystals exhibit decreased packing density and high light scattering effect, resulting in poor resolution and rather low efficiency [6].

In contradiction to the analogous bulk samples, nanoscopic phosphors provide the following advantages: (i) high packing density; (ii) low light scattering effects; (iii) shorter preparation time; (iv) lower sintering temperatures (when necessary); and (v) easy suspendability in liquid media. The latter is a decisive requirement for the application of phosphors in life science, for example as labeling materials for ultrasensitive detection of biological species such as antibodies, DNA and cells [7], and not fulfilled by microscopic luminescent materials. There is, on the other hand, still the disadvantage of lower quantum efficiency compared to bulk materials in most of the cases due to large surface/bulk ratios for small particles leading to increasing nonradiative relaxation.

In contrast to numerous papers dealing with bulk Ba_2_SiO_4_:Eu^2+^ materials very few publications about the production of submicron-sized Ba_2_SiO_4_:Eu^2+^ crystals are available. This is remarkable in view of the importance of that phosphor. In these works, the crystal sizes are generally larger than 0.5 μm, prepared by applying laborious and time-consuming methods as sol-gel synthesis, spray pyrolysis and vapor phase techniques [8,9]. Due to the low starting temperature and the short calcination time, combustion synthesis is an energy-saving method, usually applied for preparation of highly crystalline nano-compounds, e.g., complex oxide ceramics such as aluminates, ferrites and chromites [10,11,12]. The process combines an exothermic redox reaction of an oxidizer (metal nitrates, ammonium nitrate or ammonium perchlorate) and a reducing organic fuel, e.g., urea, hydrazine or glycine [10,11,12,13]. Urea is reported to act additionally as dispersive agent for the NPs [14]. Main disadvantages of the combustion method are the difficulties in scaling up the process for industrial phosphor production and the fact that it can only be performed on air. The combustion synthesis is therefore not appropriate for synthesizing air-sensitive samples.

In this work, the combustion approach was applied for the synthesis of undoped and Eu^3+^, Eu^2+^ and Sr^2+^ doped Ba_2_SiO_4_ NPs, aiming to explore the variation of the emission color and to investigate the optical properties of these nanophosphors. Here, we present small, homogeneous spherical Ba_2_SiO_4_:Eu^2+^ NPs for the first time and compared their luminescence properties with the analogous bulk phosphors. Subsequently, the doping ion Eu^2+^ was changed to Eu^3+^ to achieve a red emission color of the nanoparticles. Finally, an attempt to prepare rare-earth-free nanophosphors was performed by introducing additional lattice defects. These lattice defects were generated by partial substitution with Sr^2+^ ions. Thus, bluely luminescent Ba_2_SiO_4_:Sr^2+^ NPs were successfully produced and a mechanism for the explanation of the nature of this unusual emission is proposed, based on temperature-dependent luminescence and lifetime measurements.

To the best of our knowledge, the production of barium orthosilicate nanoparticles by means of combustion synthesis is reported in this work for the first time. In addition, the size of the Ba_2_SiO_4_ NPs produced applying the combustion synthesis is smaller than the size of the ones prepared with the aid of other synthesis methods [8,9]. Undoped barium orthosilicate nanoparticles as well as those doped with Eu^3+^ and Sr^2+^ have never been produced or investigated before.

## 2. Results and Discussion

### 2.1. X-Ray Diffraction Analysis

**Figure 1:**
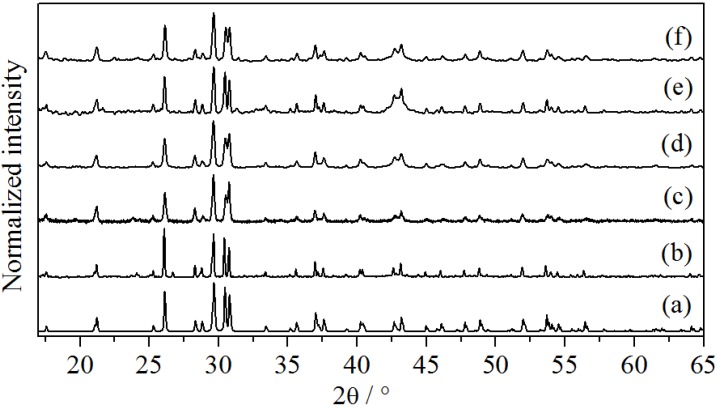
(**a**) Calculated X-ray diffraction (XRD) pattern for Ba_2_SiO_4_ [15]. XRD measurements for (**b**) synthesized bulk Ba_2_SiO_4_:Eu^2+^; (**c**) Ba_2_SiO_4_:Eu^3+^ NPs after combustion; (**d**) Ba_2_SiO_4_:Eu^2+^ NPs after annealing; (**e**) undoped Ba_2_SiO_4_ NPs and (**f**) Ba_2_SiO_4_:Sr^2+^ NPs.

Powder X-ray diffraction analysis was used for the confirmation of the crystal structure of the synthesized compounds. The results presented in the Figure 1 show the formation of pure Ba_2_SiO_4_ for all experiments and perfect match with the calculated pattern [15]. In this case, the powder diffraction pattern of the bulk sample is characterized by the high crystallinity and causes narrow-shaped reflections (Figure 1b), while the other samples present broader signals, typical for nano-sized crystals (Figure 1c–f). In Figure 1c, it is observed that the crystal structure of Ba_2_SiO_4_ is already formed after the combustion process. The post-synthesis annealing step is then performed for improving the crystallinity and/or reducing the Eu^3+^ to Eu^2+^ ions.

### 2.2. Particle Morphology and Size

**Figure 2:**
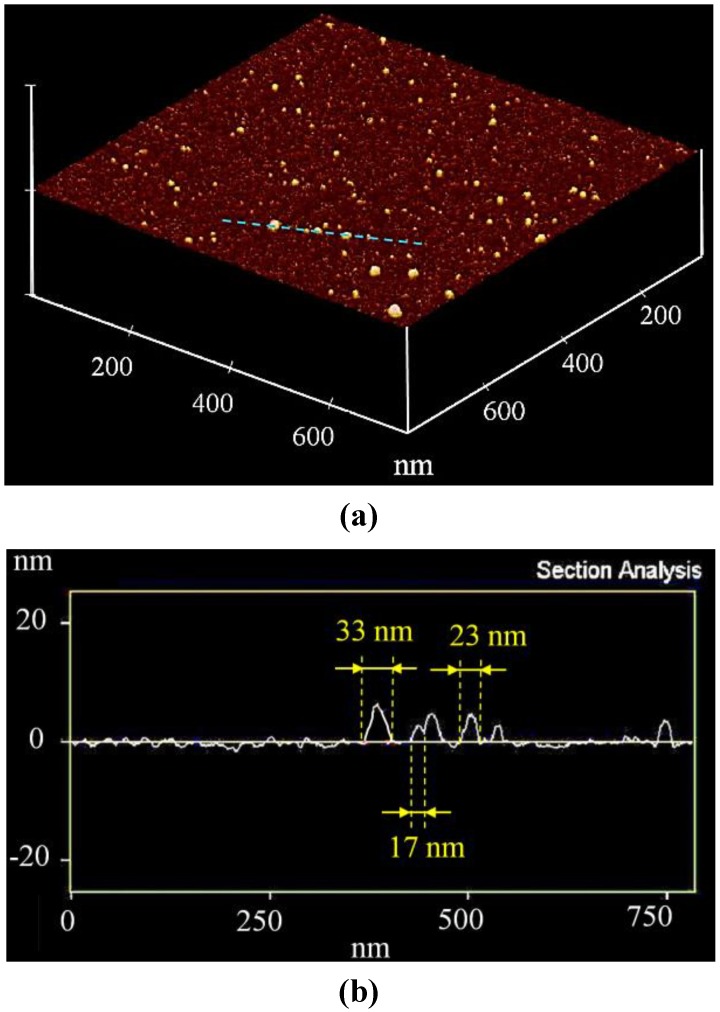
3D atomic force microscopy (AFM) image of Ba_2_SiO_4_:Eu^2+^ NPs on a mica surface (**a**); section analysis according to the blue line in the image (**b**).

The particle size of the barium orthosilicate NPs was characterized by three different methods: (i) estimations with aid of the Scherrer Equation [16]; (ii) Atomic Force Microscopy (AFM) and (iii) Scanning Electron Microscopy (SEM). From the width of the reflexes at 2θ = 21°, 26° and 28° of the X-ray diffraction analysis shown in Figure 1, the particle size of Ba_2_SiO_4_:Eu^2+^ was estimated to be about 35 nm. This estimation was confirmed by the AFM images presented in Figure 2, where the spherical morphology of the barium orthosilicate particles was observed. In addition, the section analysis of the AFM images on the position marked in blue (see Figure 2) allowed the detection of the existence of smaller particles with diameter of, e.g., 17–33 nm. Hence, the barium orthosilicate NPs produced here by means of the combustion method present superior properties in comparison to the particles previously reported in the literature, which either lie rather in the sub-micrometer range or are irregularly shaped [8,9].

**Figure 3:**
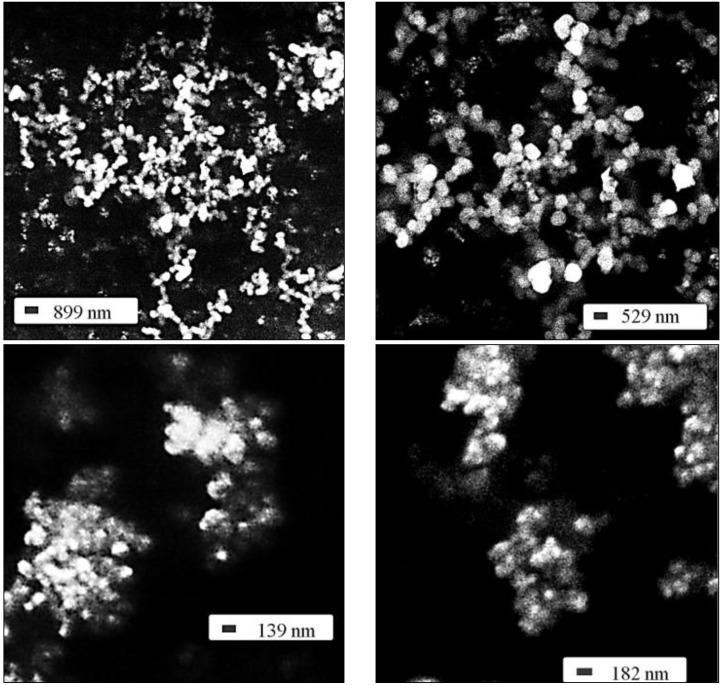
Scanning electron microscopy (SEM) images of Ba_2_SiO_4_:Eu^2+^ nanoparticles at different amplifications.

The size analysis was completed studying the SEM images presented in Figure 3. This SEM images were important for detecting the formation of particle agglomerates, which have sizes beyond the detectable range of the AFM measurements. The observed agglomerates are rather small (about 300–500 nm), probably formed by the merge of single particles during the post-synthetic annealing process and can be easily dispersed by sonication treatments.

**Figure 4:**
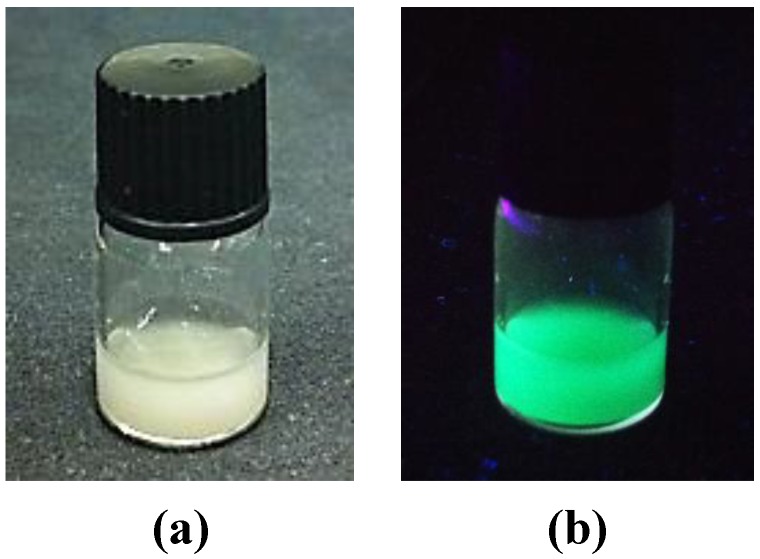
Ethanolic suspension of Ba_2_SiO_4_:Eu^2+^ nanoparticles under day (**a**) and UV light (**b**).

Due to the small size, nanocrystals are less susceptible to the influence of gravity and can be suspended in liquid media. This property is an important condition for the application of phosphors in life science and not fulfilled by the analogous bulk samples. As shown in Figure 4, the Ba_2_SiO_4_:Eu^2+^ NPs are able to form a stable suspension in ethanol, which displays a strong green luminescence upon irradiation with UV light.

### 2.3. Optical Properties

#### 2.3.1. Ba_2_SiO_4_:Eu^2+/3+^

**Figure 5:**
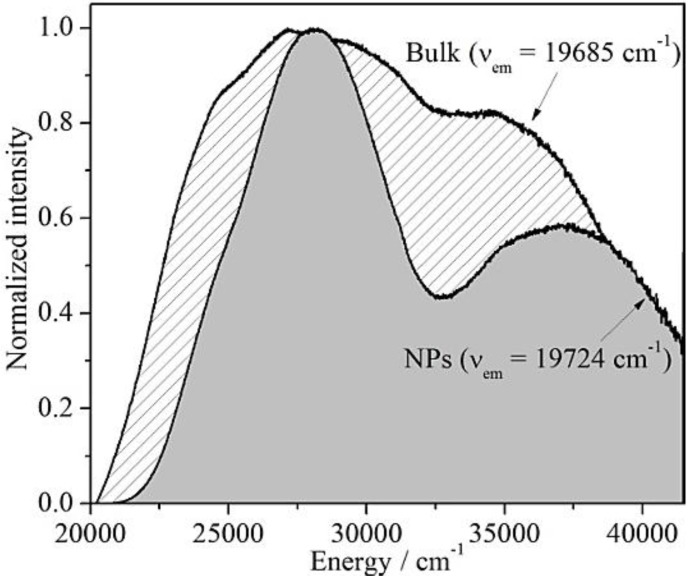
Excitation spectra of Ba_2_SiO_4_:Eu^2+^ microcrystals (ν_em_ = 19,685 cm^−1^) and nanoparticles (ν_em_ = 19,724 cm^−1^).

**Figure 6:**
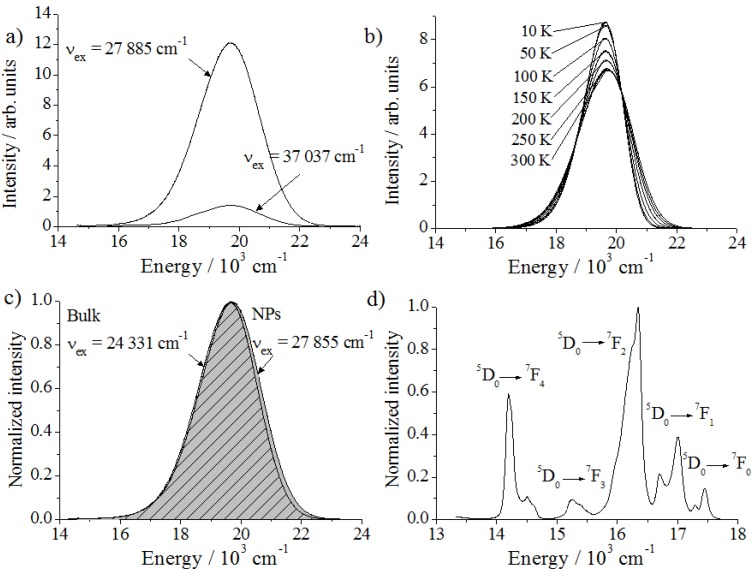
Emission spectra. (**a**) Ba_2_SiO_4_:Eu^2+^ NPs for different excitation energies and (**b**) temperature-dependent measurement of Ba_2_SiO_4_:Eu^2+^ bulk phosphor (ν_ex_ = 24,510 cm^−1^). (**c**) Comparison between Ba_2_SiO_4_:Eu^2+^ in bulk and in nano form and (**d**) Ba_2_SiO_4_:Eu^3+^ nanophosphor (ν_ex_ = 25,316 cm^−1^).

Excitation spectra of Ba_2_SiO_4_:Eu^2+^ bulk and nanophosphors are both broadly distributed over the UV spectral range between 20,000 and 42,000 cm^−1^ (Figure 5). The broader bands of the bulk material and the intensity increase around 32,000 cm^−1^ in contrast to the formation of two discrete absorption bands of the NPs may be explained by four different hypotheses: (i) the presence of an impurity phase in the bulk material, which cannot be detected by XRD; (ii) the occupation of two different Ba^2+^-sites by the Eu^2+^ ions; (iii) the crystal field splitting of the 4f^6^5d^1^ electronic configuration due to the low-symmetry coordination of the Eu^2+^ ions and (iv) a saturation effect due to the high doping concentration of 1% [17]. In order to investigate the origin of the two discrete bands on the excitation spectra, several emission measurements were performed, which are depicted in the Figure 6a,b.

In general, the emission spectrum of Ba_2_SiO_4_:Eu^2+^ consists of a broad band peaking at 19,706 cm^−1^ (Figure 6a–c), resulting from the 4f^6^5d^1^ → 4f^7^ electronic transition of Eu^2+^ in this lattice. As the optical properties of divalent europium strongly depend on the environmental conditions, the presence of an impurity phase would result in a change on the emission spectrum. Therefore, if the two discrete excitation bands in the Figure 5 would belong to two different structures, excitations of the sample at 27,855 cm^−1^ and 37,037 cm^−1^ would generate deviations of the respective emission spectra. However, the emission spectra recorded for these two excitation energies are both centered at 19,706 cm^−1^ and present similar values of full width at half maximum (FWHM) of 2403 cm^−1^ and 2413 cm^−1^ for the spectra excited at, respectively, 27,855 cm^−1^ and 37,037 cm^−1^ (Figure 6a). The maximum of the emission spectrum at 19,706 cm^−1^ is comparable to the value of 19,531 cm^−1^, described in the literature for Ba_2_SiO_4_:Eu^2+^ particles [8]. In this case, the minor deviation of approximately 200 cm^−1^ may be explained by the different doping concentrations [4] of 1% in our work and of 3% in the work of Han *et al.* [8].

As previously described in the literature [1,2,3], the barium ions occupy two different sites in the crystal structure of Ba_2_SiO_4_: one nine-fold and another ten-fold coordinated site. The occupancy of Eu^2+^ of both Ba^2+^-sites causes a slight asymmetry of the Gaussian shape of the Ba_2_SiO_4_:Eu^2+^ emission band. This is assigned to the overlap of two emission bands centered at approximately 19,200 cm^−1^ and 19,800 cm^−1^ [1,3].

Trying to improve the resolution of these two overlapped emission bands, the luminescence spectra of Ba_2_SiO_4_:Eu^2+^ were measured at temperatures varying between 10 and 300 K (Figure 6b). At 10 K, the maximum of the emission intensity is located at 19,627 cm^−1^ with a fwhm of 1552 cm^−1^. As expected, after cooling down to 10 K, an increase in the emission intensity and a decrease on the band width were observed. However, the emission band is not split. Presumably, the energetic position of the excited states related to Eu^2+^ on both Ba^2+^-sites are very similar and cannot be detected spectroscopically for the doping concentration of 1%. Furthermore, it agrees with the fact that the excitation spectra recorded for different emission energies between 16,000 cm^−1^ and 22,000 cm^−1^ remain practically invariable. Another explanation, which does not necessarily exclude the previously mentioned one, is the possible energy transfer between Eu^2+^ ions occupying two different sites, resulting in one single emission band. An enhanced interaction between Eu^2+^ ions is expected because of the applied high doping concentrations. Due to these reasons, the two absorption bands presented in the Figure 5 are also not related to the occupation of the different Ba^2+^ sites by the activator ion and are presumably associated to the crystal field splitting. A saturation effect in the bulk material owing to the high doping concentration of Eu^2+^ ions, however, could also be an explanation, which had already been discussed in literature [17]. Even though the doping concentration is equal for the nano and bulk material, the saturation effect is expected to be more pronounced manifested in the bulk Ba_2_SiO_4_:Eu^2+^ phosphor, causing the broadening on the excitation spectrum observed on Figure 5. In nanoparticles, the single Eu^2+^ ions are mostly separated by the particle boundaries, reducing the interaction with each other.

Besides understanding the origin of the emission and excitation energies, it is also important to investigate possible changes on the photoluminescence of miniaturized phosphors. A decrease on the crystal size can influence the band gap energy and, consequently, the optical properties of nanophosphors [6]. As depicted in Figure 6c, the difference between the emission spectrum of nano and bulk Ba_2_SiO_4_:Eu^2+^ is negligible. Therefore, it was possible to reduce the crystal size of the Eu^2+^-activated barium orthosilicate down to the nano-scale avoiding drastic changes on the emission and excitation energies.

**Figure 7:**
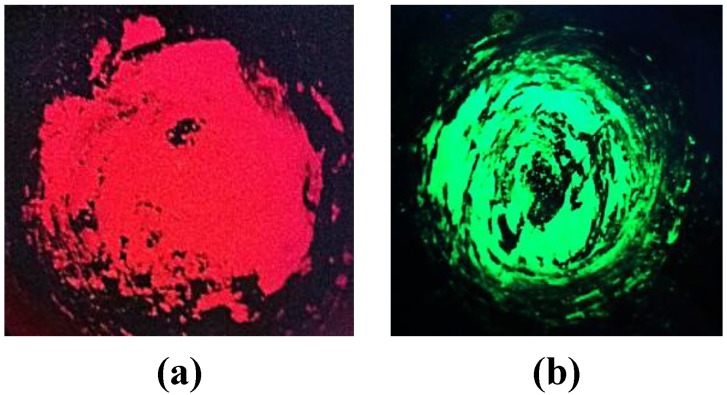
Barium orthosilicate nanoparticles doped with Eu^3+^ (**a**) and Eu^2+^ (**b**), irradiated with UV light.

**Figure 8:**
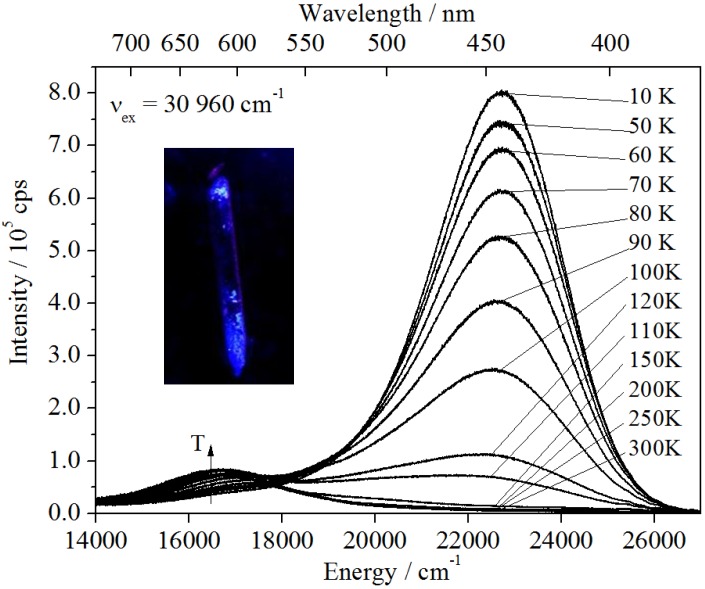
Temperature dependent emission of Ba_2_SiO_4_:Sr^2+^ NPs: decreasing maximum at 22,760 cm^−1^ and increasing maximum at 16,690 cm^−1^ for higher temperatures. Inset: Ba_2_SiO_4_:Sr^2+^ NPs under UV light, cooled down with liquid N_2_.

In addition to Ba_2_SiO_4_:Eu^2+^, undoped Ba_2_SiO_4_ as well as Ba_2_SiO_4_:Eu^3+^ and Ba_2_SiO_4_:Sr^2+^ nanoparticles were spectroscopically characterized. The undoped Ba_2_SiO_4_ NPs do not show any luminescence, while the nanophosphors doped with Eu^2+^, Eu^3+^ and Sr^2+^ emit in the green, red and blue range, respectively (Figure 7 and Figure 8a).

As expected, the emission spectrum of the Ba_2_SiO_4_:Eu^3+^ NPs consists of several peaks distributed over the red spectral range, which are assigned to the ^5^D_0_ → ^7^F*_J_* (*J* = 0–4) transitions of the trivalent europium ions (Figure 6d). The distortions and the defects in the nanoparticles also due to the large surface cause slightly different coordination spheres of the Eu^3+^ ions. Therefore, the transitions are relatively broad, and no crystal field splitting could be detected, which is generally the case for nanoparticles doped with trivalent lanthanides. It is remarkable that the transition ^5^D_0_ → ^7^F_0_ shows some intensity, which can be understood because the site symmetry is expected to be rather low. Also the ^5^D_0_ → ^7^F_4_ transition is relatively strong compared to the ^5^D_0_ → ^7^F_2_ one.

#### 2.3.2. Ba_2_SiO_4_:Sr^2+^

In the case of Ba_2_SiO_4_:Sr^2+^, two emission bands can be observed (see Figure 8b), while undoped Ba_2_SiO_4_ show no luminescence. The blue-shifted band is located at 22,321 cm^−1^ (FWHM = 3286 cm^−1^) giving rise to the strong blue luminescence of the nanoparticles at low temperatures (Figure 8a). It is assigned to the presence of self-trapped excitons (STEs) in the nanoparticles (see below). However, it is totally quenched at temperatures above 120–130 K. At that temperature, another emission band located at 16,643 cm^−1^ arises, which increases in intensity with increasing temperature. Thus, a correlation between these two bands is assumed, as will be discussed in more detail below.

**Figure 9:**
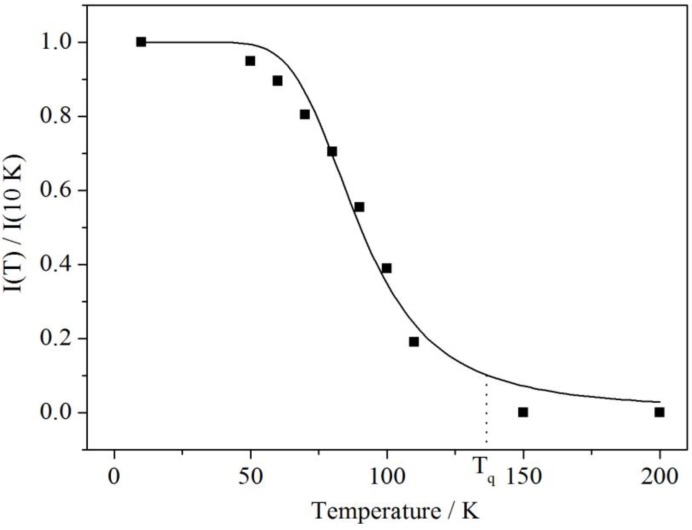
Temperature dependence of the normalized integrated luminescence intensity of the blue-shifted emission in Ba_2_SiO_4_:Sr^2+^. The black curve was fitted according to Equation (1) with *C* = 616 and *E_a_* = 50 meV or 400 cm^−1^.

Figure 9 shows the temperature dependence of the integrated intensity of the blue emission band. It can be fitted to the well-known Mott Equation (1) [18]

(1) $$I\left(T\right)=\;\frac{I_{0}}{\left[1+C\cdot \exp(-E_{a}/k_{B}T)\right]}$$

where *C* is the ratio between the thermal quenching rate and the radiative decay rate (*C* = *p*/*k*_rad_) and *E_a_* is the activation barrier for the non-radiative process.

From the fit of the temperature dependent measurements the values *C* = 616 and *Ea=* 50 meV or 400 cm^−1^ are obtained, respectively. The large value of C indicates a more efficient quenching rate, which is in agreement with the relatively low temperature of *T*_q_(*I*(*T*_q_)/*I*_0_ = 0.1) ≈ 120–130 K. At higher temperatures the high-energy band is quenched very efficiently. On the other hand, the activation energy is in a similar range to the energies of the vibrational modes of the SiO_4_^4−^ tetrahedron, which have been reported by Handke and Urban for Ba_2_SiO_4_ to be roughly 350 cm^−1^ for the E bending mode and 490–520 cm^−1^ for the T_2_ bending mode [19]. Furthermore, as studied for SiO_2_, the structural nature of the STE in silicates is solely based on the SiO_4_^4−^ tetrahedron and consists of a peroxy linkage (Si–O–O) with two holes in a σ_u_ bond of an O_2_ molecule and two electrons trapped in an oxygen vacancy ($V_{O}^{x}$) [20]. Thus, a thermally induced activation of electrons via coupling to the vibrational modes of the silicate anion is expected. Moreover, this interpretation makes our assignment of the blue-shifted band clearly reasonable since the spectral position coincides with results found for SiO_2_, which was reported to show an emission at 2.8 eV (22,582 cm^−1^) [20,21]. A similar temperature behavior was also found for that band totally quenching in the range between 110 and 120 K [22]. However, in these nanoparticles, the stabilization of the excitons arises from the presence of the Sr^2+^ ions, which induce distortions large enough to trap the excitons. This also explains why no such emission is observed in the case of undoped Ba_2_SiO_4_.

**Figure 10:**
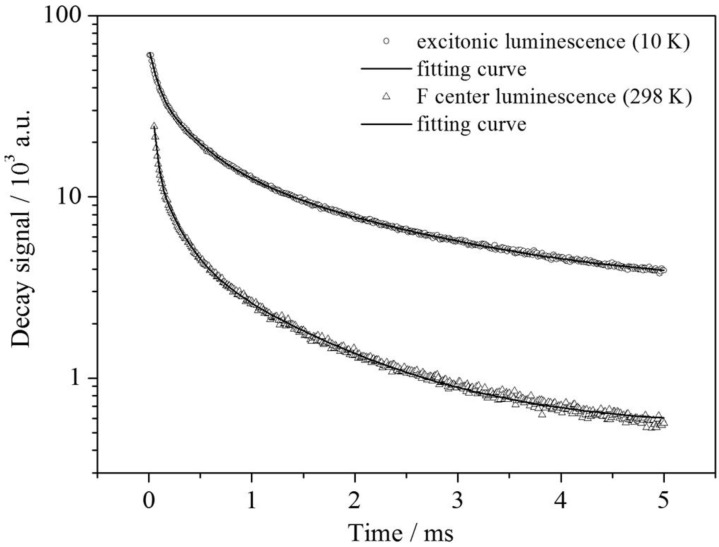
Semi-log plot of the luminescence decays of the observed emission bands in Ba_2_SiO_4_:Sr^2+^. The black curves correspond to tri-exponential fits, respectively. For the values: see text.

In order to investigate other intrinsic characteristics of the excitonic emission, which are also useful for the interpretation, lifetime measurements at 10 K and room temperature have been performed. The luminescence decay of the blue emission shows a tri-exponential behavior (see Figure 10) with the lifetimes *τ*_1_ = 1.76 ms (22%), *τ*_2_ = 0.36 ms (36%) and *τ*_3_ = 0.07 ms (41%). The first value is interpreted as the radiative decay of the STE and in good agreement with the value found for self-trapped excitons in SiO_2_ (~1 ms) [23,24,25]. The other two lifetimes already indicate trapping processes at the Sr^2+^ sites inducing a distortion that is obviously necessary to stabilize the excitons.

**Figure 11:**
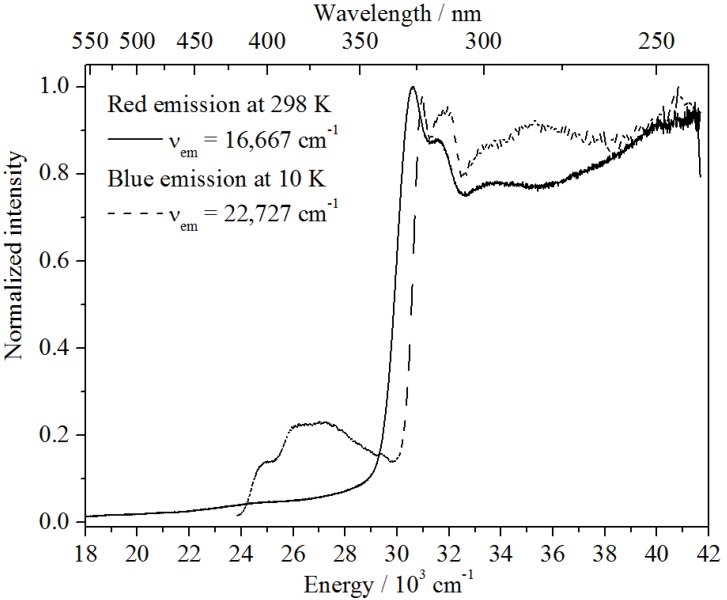
Excitation spectra of the red emission band at 10 K (bold curve) and of the blue emission band at 298 K (dashed curve).

For an understanding of the origin of the red-shifted band at 16,643 cm^−1^, one has to include both the temperature behavior as well as the lifetime measurements. Since that emission shows an increase just once the blue-shifted emission has been thermally quenched, a deep correlation between these two centers is supposed. This also becomes clear from the excitation spectra depicted in Figure 11. Upon detection of the red emission at room temperature, a very similar excitation spectrum to the excitonic emission at 10 K is obtained. The slight redshift can be explained by the temperature difference. Moreover, the luminescence decay of the color center detected at room temperature is very similar to the decay of the excitonic emission at 10 K (see Figure 10). As in the upper case, a tri-exponential fit can be performed affording the lifetimes, *τ*_1_ = 1.15 ms (8%), *τ*_2_ = 0.21 ms (18%) and *τ*_3_ = 0.03 ms (74%). The deviations can be explained by the shortening of the lifetimes due to non-radiative relaxation.

**Figure 12:**
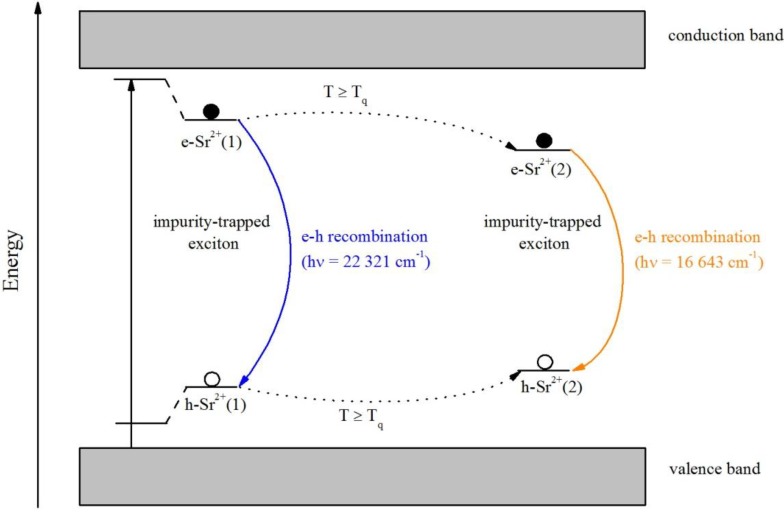
Proposed model for the electron transfer between the valence and conduction band in Sr^2+^-doped Ba_2_SiO_4_ NPs.

Thus, we assume that after heating at temperatures higher than *T*_q_, the excitons located at the Sr^2+^ impurities become mobile and move within the lattice until they become trapped at another Sr^2+^ site due to the induced distortion. A pictorial representation of the model is depicted in Figure 12. During the movement, they lose energy non-radiatively, which gives rise to a larger Stokes shift and therefore a red-shift of the emission. Therefore, *T*_q_ can be interpreted as the temperature at which the excitons become mobile. After trapping at another Sr^2+^ site, the intensity decreases with increasing temperature via multiphonon relaxation. The similarity of the two centers is indicated by the similarity of the excitation spectra and the decay curves. Photoconductivity and thermoluminescence measurements could still clarify whether an involvement of the conduction band in the mechanism is the case.

## 3. Experimental Section

All reagents were commercially purchased and used without further purification. For the preparation of undoped Ba_2_SiO_4_ nanoparticles, Ba(NO_3_)_2_ (Merck Chemicals, Darmstadt, Germany, 99+%), Si(OC_2_H_5_)_4_ (Alfa Aesar, Karlsruhe, Germany, 98%), H_3_BO_3_ (Acros Organics, Geel, Belgium, 99+%) and NH_2_CONH_2_ (Alfa Aesar, 99+%) were applied as starting materials. In this case, H_3_BO_3_ was added as a flux medium and NH_2_CONH_2_ as fuel for the combustion synthesis [26]. In typical experiments, the stoichiometric amount of barium nitrate was dissolved in 10 mL of distilled water together with urea and boric acid, resulting in a molar ratio of Ba^2+^:NH_2_CONH_2_:H_3_BO_3_ of 1:10:0.06. In parallel, the doubled stoichiometric amount of tetraethyl orthosilicate (TEOS) was dissolved in 2 mL of ethanol. The solution in ethanol causes the hydrolysis of TEOS to Si(OH)_4_. The excess is required to overcome the rapid TEOS evaporation. Afterwards, the ethanolic solution is added drop wise to the aqueous solution under vigorous stirring. The solution was subsequently transferred to a pre-heated muffle furnace at 600 °C. At that temperature, the solution evaporates, generating a large amount of gases as oxides of carbon and nitrogen. The organic fuel causes the combustion, which releases the energy necessary for the synthesis of the nanopowder. The reaction is completed after about 5 to 10 min. Doping the nanomaterials with 1% Sr^2+^ and 1% Eu^3+^ was achieved by adding the respective amounts of Sr(NO_3_)_2_ (Merck, p.a.) or Eu(NO_3_)_3_ (Chempur, Karlsruhe, Germany, 99.9%) to the aqueous solution, respectively. After the combustion, the samples were calcined at 1000 °C for several hours. Doping of Eu^2+^ has been achieved by performing the calcination of the Eu^3+^ doped materials under a H_2_/Ar reductive atmosphere.

The bulk material Ba_2_SiO_4_:Eu^2+^ was prepared for comparison of the optical properties with those of the analogous nanophosphor in a typical solid-state reaction according to the Equation [14]:

2BaO_2_ + SiO_2_ → Ba_2_SiO_4_ + O_2_ (2)

For this reason, stoichiometric amounts of BaO_2_ and SiO_2_ were ground together for 1 h and calcined under N_2_ atmosphere. The temperature program for the calcination was carried out applying the following heating steps: (1) 200 °C/h to 400 °C; (2) 25 °C/h to 450 °C for 20 min; (3) 25 °C/h to 500 °C and (4) 150 °C/h to 1050 °C, holding for 20 h. As a last step, the freshly prepared host compound was mixed with 1% EuO under argon atmosphere and further calcined at 1000 °C. EuO was prepared by mixing stochiometric amounts of Eu_2_O_3_ and Eu metal and heating the starting materials at 1200 °C for two weeks in a tantalum container, which was coated with a silica ampoule. In this context, it is mentionable that our method of doping with Eu^2+^ ions is rather uncommon, since most of the articles describe doping with Eu_2_O_3_ followed by reduction [4]. Direct doping with Eu^2+^ ions is, however, much more advantageous in order to avoid defects, which might be created during the reduction process.

Photoluminescence measurements were performed with aid of a Fluorolog3 spectrofluorometer Fl3-22 (Horiba Jobin Yvon, Longjumeau, France) equipped with double Czerny-Turner monochromators, a 450 W xenon lamp and a R928P photomultiplier (Hamamatsu, Herrsching, Germany) with a photon-counting system. Cooling down to 10 K was achieved by a closed cycle He cryostat (Janis Research, Wilmington, United States). Lifetime measurements have been performed using a Xe-flashlamp (Ushio Inc., Tokyo, Japan) with a time resolution down to 10 μs. Powder X-ray diffraction was measured on a D5000 X-ray diffractometer (Siemens, Karlsruhe, Germany) operating at 40 kV, 30 mA with Cu-Kα radiation (λ = 0.154178 nm). Microscopy analysis was carried out on a CS44 Scanning Electron Microscope (SEM, CamScan, Cambridge, UK) and on an Atomic Force Microscope (AFM) Multimode 2, applying an AC200TS cantilever.

## 4. Conclusions

The energy and time-saving combustion synthesis was flexibly applied for the production of doped and undoped barium orthosilicate nanoparticles leading to very small nanoparticles of sizes of about 35 nm. By means of different approaches, red-green-blue luminescence was obtained doping the Ba_2_SiO_4_ NPs respectively with Eu^3+^, Eu^2+^ and Sr^2+^. Partial substitution of the Ba^2+^ ions with Sr^2+^ ions afforded lattice defects, which are stabilized by the *in situ* lattice imperfections of the Ba_2_SiO_4_ nanocrystals. In this way, rare-earth-free blue luminescence of the Ba_2_SiO_4_ nanoparticles could be generated for the first time. Emission spectra of the Ba_2_SiO_4_:Eu^2+^ NPs consist of a broad band centered at 19,706 cm^−1^ (507 nm), assigned to the 4f^6^5d^1^ → 4f^7^ electronic transition of the divalent europium and do not significantly differ from the analogous bulk phosphor. In the case of Ba_2_SiO_4_:Eu^3+^ NPs, the typical ^5^D_0_ → ^7^F*_J_* (*J* = 0–4) transitions of Eu^3+^ are observed in the emission spectrum. Furthermore, the nature of the defect luminescence on Ba_2_SiO_4_:Sr^2+^ NPs was investigated by means of temperature-dependent UV/VIS spectroscopy and lifetime measurements. It was attributed to the presence of impurity-trapped excitons, which become trapped at the Sr^2+^ that cause distortions in the lattice thus stabilizing the excitons. At temperatures above *T*_q_ = 120–130 K, the blue emission is efficiently quenched whereas an orange emission at 16,643 cm^−1^ (600 nm) suddenly occurs. A model to explain this untypical behavior is suggested, which is in agreement with the temperature dependence and the similarity of the decay curves and excitation spectra of the observed emissions. It includes a thermally activated motion of the impurity-trapped excitons to another Sr^2+^ site in the lattice, which involves non-radiative relaxation thus leading to a red-shift of the emission. Photoconductivity and thermoluminescence measurements would, however, help to clarify an involvement of the conduction band in the proposed mechanism. Supplementary, the Scherrer equation was combined with SEM and AFM images for the characterization of the particle size. Finally, it was demonstrated that the produced Ba_2_SiO_4_:Eu^2+^ NPs are able to build stable suspensions in liquid media, presenting green luminescence, when irradiated with UV light.
